# Rapid Sequencing of the Bamboo Mitochondrial Genome Using Illumina Technology and Parallel Episodic Evolution of Organelle Genomes in Grasses

**DOI:** 10.1371/journal.pone.0030297

**Published:** 2012-01-17

**Authors:** Peng-Fei Ma, Zhen-Hua Guo, De-Zhu Li

**Affiliations:** 1 Key Laboratory of Biodiversity and Biogeography, Kunming Institute of Botany, Chinese Academy of Sciences, Kunming, Yunnan, People's Republic of China; 2 Plant Germplasm and Genomics Center, Germplasm Bank of Wild Species, Kunming Institute of Botany, Chinese Academy of Sciences, Kunming, Yunnan, People's Republic of China; 3 Graduate University of Chinese Academy of Sciences, Beijing, People's Republic of China; J. Craig Venter Institute, United States of America

## Abstract

**Background:**

Compared to their counterparts in animals, the mitochondrial (mt) genomes of angiosperms exhibit a number of unique features. However, unravelling their evolution is hindered by the few completed genomes, of which are essentially Sanger sequenced. While next-generation sequencing technologies have revolutionized chloroplast genome sequencing, they are just beginning to be applied to angiosperm mt genomes. Chloroplast genomes of grasses (Poaceae) have undergone episodic evolution and the evolutionary rate was suggested to be correlated between chloroplast and mt genomes in Poaceae. It is interesting to investigate whether correlated rate change also occurred in grass mt genomes as expected under lineage effects. A time-calibrated phylogenetic tree is needed to examine rate change.

**Methodology/Principal Findings:**

We determined a largely completed mt genome from a bamboo, *Ferrocalamus rimosivaginus* (Poaceae), through Illumina sequencing of total DNA. With combination of *de novo* and reference-guided assembly, 39.5-fold coverage Illumina reads were finally assembled into scaffolds totalling 432,839 bp. The assembled genome contains nearly the same genes as the completed mt genomes in Poaceae. For examining evolutionary rate in grass mt genomes, we reconstructed a phylogenetic tree including 22 taxa based on 31 mt genes. The topology of the well-resolved tree was almost identical to that inferred from chloroplast genome with only minor difference. The inconsistency possibly derived from long branch attraction in mtDNA tree. By calculating absolute substitution rates, we found significant rate change (∼4-fold) in mt genome before and after the diversification of Poaceae both in synonymous and nonsynonymous terms. Furthermore, the rate change was correlated with that of chloroplast genomes in grasses.

**Conclusions/Significance:**

Our result demonstrates that it is a rapid and efficient approach to obtain angiosperm mt genome sequences using Illumina sequencing technology. The parallel episodic evolution of mt and chloroplast genomes in grasses is consistent with lineage effects.

## Introduction

Next-generation sequencing that is not only high-throughput but also low-cost has already revolutionized approaches for genome sequencing and is now becoming ‘now-generation’ sequencing [1]. Recently complete or nearly complete chloroplast (cp) genomes of plants have been successfully recovered by Illumina sequencing or 454 pyrosequencing from total DNA containing cpDNA as well as nuclear and mitochondrial (mt) DNA [2]–[4]. Compared to conventional approaches for cp genome sequencing involving purification or PCR amplification of cpDNA [5], these methods are more simple and effective [2]–[4]. On the contrary, the next-generation sequencing technologies are just beginning to be applied to sequencing of mt genomes of plants and only a few examples of plant mt genome next-generation sequencing have been published so far [6]–[8]. The 21 available mt genomes of higher plants (Table 1) are essentially obtained by Sanger sequencing, involving extracting or PCR amplification of mtDNA and library construction prior to sequencing. This time- and labour-intensive sequencing approach to some extent limits the sequencing of plant mt genomes. Recently, we have sequenced six bamboo cp genomes from total DNA that was enriched for cpDNA using Illumina sequencing [9]. Meanwhile, we found a large number of sequence reads in the total reads being of mtDNA origin. Could the whole or largely completed mt genome be assembled from these reads at the same time? And if so, this would be a rapid and efficient approach to sequence angiosperm mt genomes and more sequenced genomes will be helpful in understanding the extraordinary evolutionary history of angiosperm mt genomes.

**Table 1:** Mitochondrial genomes of the 22 seed plants included in phylogenetic analyses in this study.

Classification	Taxa	Accession number	Reference
Gymnosperms			
	*Cycas taitungensis*	NC_010303	Chaw et al., 2008 [65]
Angiosperms			
Monocots	*Bambusa oldhamii*	EU365401	Lin et al., unpublished
	*Ferrocalamus rimosivaginus*	JQ235166–JQ235179	Current study
	*Oryza rufipogon*	NC_013816	Fujii et al., 2010 [6]
	*Oryza sativa*	NC_011033	Notsu et al., 2002 [38]
	*Triticum aestivum*	NC_007579	Ogihara et al., 2005 [39]
	*Tripsacum dactyloides*	NC_008362	Allen et al., unpublished
	*Sorghum bicolor*	NC_008360	Allen et al., unpublished
	*Zea luxurians*	NC_008333	Allen et al., unpublished
	*Zea mays*	NC_007982	Clifton et al., 2004 [66]
	*Zea perennis*	NC_008331	Allen et al., unpublished
Eudicots	*Arabidopsis thaliana*	NC_001284	Unseld et al., 1997 [67]
	*Beta vulgaris*	NC_002511	Kubo et al., 2000 [68]
	*Brassica napus*	NC_008285	Handa, 2003 [69]
	*Carica papaya*	NC_012116	Rice et al., unpublished
	*Citrullus lanatus*	NC_014043	Alverson et al., 2010 [49]
	*Cucurbita pepo*	NC_014050	Alverson et al., 2010 [49]
	*Nicotiana tabacum*	NC_006581	Sugiyama et al., 2005 [70]
	*Ricinus communis*	NC_015141	Rivarola et al., unpublished
	*Silene latifolia*	NC_014487	Sloan et al., 2010 [71]
	*Vigna radiata*	NC_015121	Alverson et al., 2011 [51]
	*Vitis vinífera*	NC_012119	Goremykin et al., 2008 [53]

The mt genomes of angiosperms exhibit a number of unique features, which distinguish them from their counterparts in animals or other organisms. These features include expanded genome size, frequent structure rearrangement via recombination, ongoing gene loss and transfer to the nuclear genome, uptake of cpDNA and nuclear DNA, and a generally low rate of molecular evolution [10]–[14]. Among them, the slow mt sequence evolution is probably the most prominent and has long been appreciated [10], [13], [14]. However, recent studies based on one or a few genes have identified several cases of rate acceleration in mt genomes of certain angiosperm lineages [15]–[19], and some of these rate increases are temporary with rates returning to normally low levels after acceleration [17], [18]. These changes in evolutionary rate are mostly restricted to mt genome as expected under locus-specific effects without correlated rate changes in cp and/or nuclear genes [15]–[19]. Nevertheless, a few studies have reported parallel rate changes in mt and cp genomes as expected under lineage effects [20], [21]. Among them a widely cited case demonstrated a faster rate of synonymous substitutions, which was correlated across cp, mt and nuclear loci in grasses relative to palm [21]. In addition, Zhong et al. [22] found that episodic rate acceleration of cp genomes occurred in the ancestral grasses and then the rate reverted to the slow rate typical of most monocot species in the descendant lineages. Here, we are interested to investigate whether the similar pattern of rate change occurred in mt genomes during grass evolution.

A well-supported phylogenetic tree that is time calibrated is necessary to examine absolute substitution rates. Although the backbone phylogeny of angiosperms has been established [23]–[25] and the major phylogenetic relationships within the grass family (Poaceae) are resolved with the whole family divided into several basal lineages plus two major lineages (the BEP clade and the PACMAD clade, core Poaceae) [9], [26]–[28], these studies have relied mainly on the cpDNA sequences. However, the mtDNA sequences are also useful for reconstructing phylogeny of angiosperms, especially at deep level [29], [30]. It would be informative to use signals from the mt genome to evaluate independently these relationships derived from the cp genome. In addition, there are numerous studies that could provide reliable estimate of divergence times during the grass evolution [25], [31]–[34]. Well-resolved phylogenetic relationships and reliable calibration in combination would make it feasible to examine the rate change in mt genomes of grasses.

Here we demonstrated a new approach for sequencing angiosperm mt genome using Illumina sequencing-by-synthesis technology and determined a largely completed mt genome from a bamboo, *Ferrocalamus rimosivaginus* T. H. Wen (Poaceae). A phylogenetic tree including 22 taxa whose mt genomes have been sequenced was reconstructed based on the sequences of 31 mt genes and the topology was nearly congruent with that inferred from the cp genome. By examining the evolutionary rate of grass mt genomes along the time-calibrated tree, we found a parallel rate change in mt and cp genomes of grasses.

## Results

### Illumina Sequencing, Genome Assembly and PCR Validation

The template DNA for the *F. rimosivaginus* cp genome sequencing, which was extracted by a rapid and simple procedure from fresh leaves, in fact contained mtDNA as well [9]. We employed a whole-genome shogun sequencing strategy and one paired-end library for Illumina sequencing was constructed with insert size of about 500 bp. The Illumina system produced 1,594,119 usable paired-end (73 bp and 75 bp) reads in one run for genome assembly [9]. These reads were a mixture of reads derived from cp, mt and nuclear genomes.

We assembled the raw reads using the software SOAPdenovo [35] with optimal parameters. All the assembled scaffolds and contigs larger than 100 bp were first mapped to the reference mt genomes from nine species of the grass family (Table 1), resulting in 68 mapped contigs and scaffolds. Subsequently we searched these 68 contigs and scaffolds for sequences with significant identity (≥90%) to the cp genome of *F. rimosivaginus*, and the aligned 3 scaffolds and 35 contigs were removed to avoid the impact of the cpDNA reads on our assembly. In addition, a 3,399 bp sequence located at one end of a scaffold and related to the remaining sequence by an estimated 386 bp length gap was deleted from this scaffold for the same reason. At this point, we obtained 16 scaffolds and 14 contigs with an N50 size of 49.6 kb, achieving a total length of 431.7 kb (Table 2, initial assembly) (see details in Table S1). The average sequencing depth was 39.5× (114,891 mt-derived paired-end reads and 7.2% of total), and there was a relatively narrow variation in the 16 scaffolds sequencing coverage, ranging from 31.9× to 46.5× with a median value of 39.3×. Although these scaffolds contained internal gaps as paired-end information was used to join contigs into scaffolds, the total number of gaps was only 24 with a mean estimated size of 151.5 bp (Table 2).

**Table 2:** Summary of the *F. rimosivaginus* mitochondrial genome sequencing and assembly.

	Initial assembly	Final assembly
Total paired-end reads	1,594,119	1,594,119
Aligned reads	114,891	114,891
Aligned (%)	7.2	7.2
Sanger reads	0	62
Total length of assembly	431,695 bp	432,839 bp
N50 value of assembly	49,579 bp	53,068 bp
Mean coverage	39.5	39.4
Number of contigs and scaffolds	30	14
Maximum contig and scaffold length	73,254 bp	93,563 bp
Number of gaps within scaffolds	24	0
Mean length of gaps	151.5 bp	n/a
Number of closed gaps	0	23
Mean length of closed gaps	n/a	172.8 bp

We further joined the initially assembled contigs and scaffolds into larger scaffolds using the sequence overlap information between them and synteny between the assembled sequences and the reference genomes. This procedure combined 4 scaffolds and 13 contigs in total. All the linkages between them were successfully confirmed by PCR amplification with conventional Sanger sequencing. To close the intra-scaffold gaps, we designed PCR primers and Sanger sequenced the amplified regions. In sum, 23 gaps (an assumed 17 bp gap was proved to be zero-length by PCR analysis and thus not accounted) were closed with a mean size of 172.8 bp which was slightly larger than the estimated size, thereby validating the linkages between regions spanning gaps within scaffolds. The final assembly had 13 scaffolds and one contig with an N50 size of 53.1 kb and a total length of 432.8 kb (Table 2, see details in Table S1), achieving an average sequencing depth of 39.4×.

Validation of linkages and closing gaps by conventional sequencing altogether generated 23,053 bp sequences, of which 16,910 bp sequences could be directly compared to the assembly for accuracy (Table S2). In this comparison, we found 21 mismatched sites distributed in 5 Sanger reads, three of which had low sequencing quality scores. Furthermore, we PCR-amplified and resequenced 16 randomly chosen regions surrounding putative genome rearrangements in comparison with the bamboo *Bambusa oldhamii* mt genome (EU365401). All the regions were successfully recovered and only one nucleotide substitution was observed in 10,837 bp resequenced regions (Table S2). In total, we tested 27,747 bp sequences by conventional sequencing, validating the accuracy of sequencing and assembly of our mt genome. Only 22 nucleotide substitution errors were found and the error rate was 0.079%, or 0.018% without accounting the errors associated with low Sanger sequence quality. This rate was close to the 0.037–0.056% error rates with the next-generation sequencings reported before [36], [37].

### Genome Features

The assembled sequence amounted to 432,839 bp distributed in 13 scaffolds and one contig. Since no estimated size existed for the *F. rimosivaginus* mt genome in previous studies, we evaluated the degree of sequence completion by comparing the assembly size to the average size (484,329 bp) of mt genomes from three closely related species *B. oldhamii*, *Oryza sativa* (NC_011033) [38] and *Triticum aestivum* (NC_007579) [39]. Based on this comparison, we could assume that the *F. rimosivaginus* mt genome has been largely assembled and 14 gaps of unknown size remained while mt genome was considered as a circular molecule [12]. The contig and scaffold sequences were deposited in GenBank under Accession Numbers JQ235166 to JQ235179.

The largely completed *F. rimosivaginus* mt genome has a GC content of 44.1% (Table 3), which is close to the median value of fully sequenced angiosperm mt genomes. As in other angiosperms [10], [12], most of the *F. rimosivaginus* mt genome sequences are noncoding sequences. The coding and intron sequences comprise only 8.9% (38,642 bp) and 5.7% (24,730 bp) of the total length, respectively, including 34 protein, 19 tRNA, and 3 rRNA genes (Table 3). These annotated genes are scattered throughout 12 of the 13 assembled scaffolds. Eight protein genes contain 22 groupII introns in all, 6 of which are *trans*-spliced.

**Table 3:** Main features of the assembled *F. rimosivaginus* mitochondrial genome.

Size of the assembly	432,839 bp
Overall GC content (%)	44.1
Protein coding genes	34
Cis-spliced groupII introns	16
Tras-spliced groupII introns	6
tRNA genes	19
rRNA genes	3
Total length of coding sequences	38,642 bp
Total length of cis-spliced introns	24,730 bp

The *F. rimosivaginus* mt genome has nearly the same coding capability as those of other grasses (Table 4). All respiratory genes except for *sdh3* and *sdh4* are present in the genome, in agreement with the suggested frequent losses of these two genes during angiosperm evolution [11], [40]. The other 15 frequently lost genes are all ribosomal protein coding genes [11], [40], among which five genes *rpl2*, *rps8*, *rps10*, *rps11*, and *rps14* are absent or appear to be pseudogenes in the sequenced mt genome. Of this group, the *rpl2* gene is the most intact with just a single 165 bp insertion compared to annotated *rpl2* gene in other grass mt genomes. Although the insertion does not alter the downstream reading frame, there is a premature stop codon in the sequence of the *rpl2* gene and thus it may be not functional. Like other grass mt genomes [41], a nearly full-length *rpl14* pseudogene is retained in the genome and the open reading frame is disrupted by several frameshift mutations. The other three genes have little or no remnant in the genome. The *F. rimosivaginus* mt genome does not have the translational capacity to recognize all the 61 sense codons even if assuming that U in the third codon/first anticodon wobble position can recognize all bases in the mtDNA, encoding only 14 of the 20 amino acids. Additionally, nearly half of the identified tRNA genes (9 out of 19) are of cp origin, and one of these genes, *trnS* (*gga*), has two copies in the genome. All three ribosomal RNA genes (*rrn5*, *rrn18*, and *rrn26*) are present in the mt genome of *F. rimosivaginus* as other grasses.

**Table 4:** Comparison of gene content among grass mitochondrial genomes.

	*Ferrocalamus rimosivaginus*	*Bambusa oldhamii*	*Triticum aestivum*	*Oryza rufipogon*	*Oryza sativa*	*Sorghum bicolor*	*Tripsacum dactyloides*	*Zea luxurians*	*Zea perennis*	*Zea mays*
Complex I										
*nad1,2,3,4, 4L,5,6,7,9*	+	+	+	+	+	+	+	+	+	+
Complex II										
*sdh3, 4*	−	−	−	−	−	−	−	−	−	−
Complex III										
*cob*	+	+	+	+	+	+	+	+	+	+
Complex IV										
*cox1,2,3*	+	+	+	+	+	+	+	+	+	+
Complex V										
*atp1,4,6,8,9*	+	+	+	+	+	+	+	+	+	+
Cytochrome c biogenesis										
*ccmB,C,FC,FN*	+	+	+	+	+	+	+	+	+	+
Ribosomal										
*rpl2*	ψ	Ψ	−	+	+	−	−	−	−	−
*rpl5*	+	+	+	+	+	−	−	−	−	−
*rpl16*	+	+	+	+	+	+	+	+	+	+
*rps1,2,3,4,7,12,13*	+	+	+	+	+	+	+	+	+	+
*rps14*	ψ	Ψ	ψ	ψ	ψ	−	−	−	−	−
*rps19*	+	+	ψ	+	+	−	−	−	−	−
Other ORFs										
*matR*	+	+	+	+	+	+	+	+	+	+
*mttB*	+	+	+	+	+	+	+	+	+	+
rRNA genes										
*rrn5,18,26*	+	+	+	+	+	+	+	+	+	+
tRNA genes of mt origin										
*trnD(guc)*	+	+	+	+	+	+	+	+	+	+
*trnE(uuc)*	+	+	+	+	+	+	+	+	+	+
*trnI(cau)*	+	+	+	+	+	+	+	+	+	+
*trnK(uuu)*	+	+	+	+	+	+	+	+	+	+
*trnfM(cau)*	+	+	+	+	+	+	+	+	+	+
*trnP(ugg)*	+	+	+	+	+	+	+	+	+	+
*trnQ(uug)*	+	+	+	+	+	+	+	+	+	+
*trnS(gcu)*	+	+	+	+	+	+	+	+	+	+
*trnS(uga)*	+	+	+	+	+	+	+	+	+	+
*trnY(gua)*	+	+	+	+	+	+	+	+	+	+
tRNA genes of cp origin										
*trnC(gca)*	+	+	+	+	+	+	+	+	+	+
*trnF(gaa)*	+	+	+	+	+	+	+	+	+	+
*trnH(gug)*	+	+	−	+	+	+	+	+	+	+
*trnM(cau)*	+	+	+	+	+	+	+	+	+	+
*trnN(guu)*	+	+	+	+	+	+	+	+	+	+
*trnP(ugg)*	+	+	−	+	+	+	+	+	+	−
*trnS(gga)*	+	+	+	+	+	+	−	−	−	−
*trnW(cca)*	+	+	+	+	+	+	+	+	+	−

+, presence of the gene; −, absence of the gene; ψ, pseudogene;

### Phylogenetic Analyses

**Figure 1:**
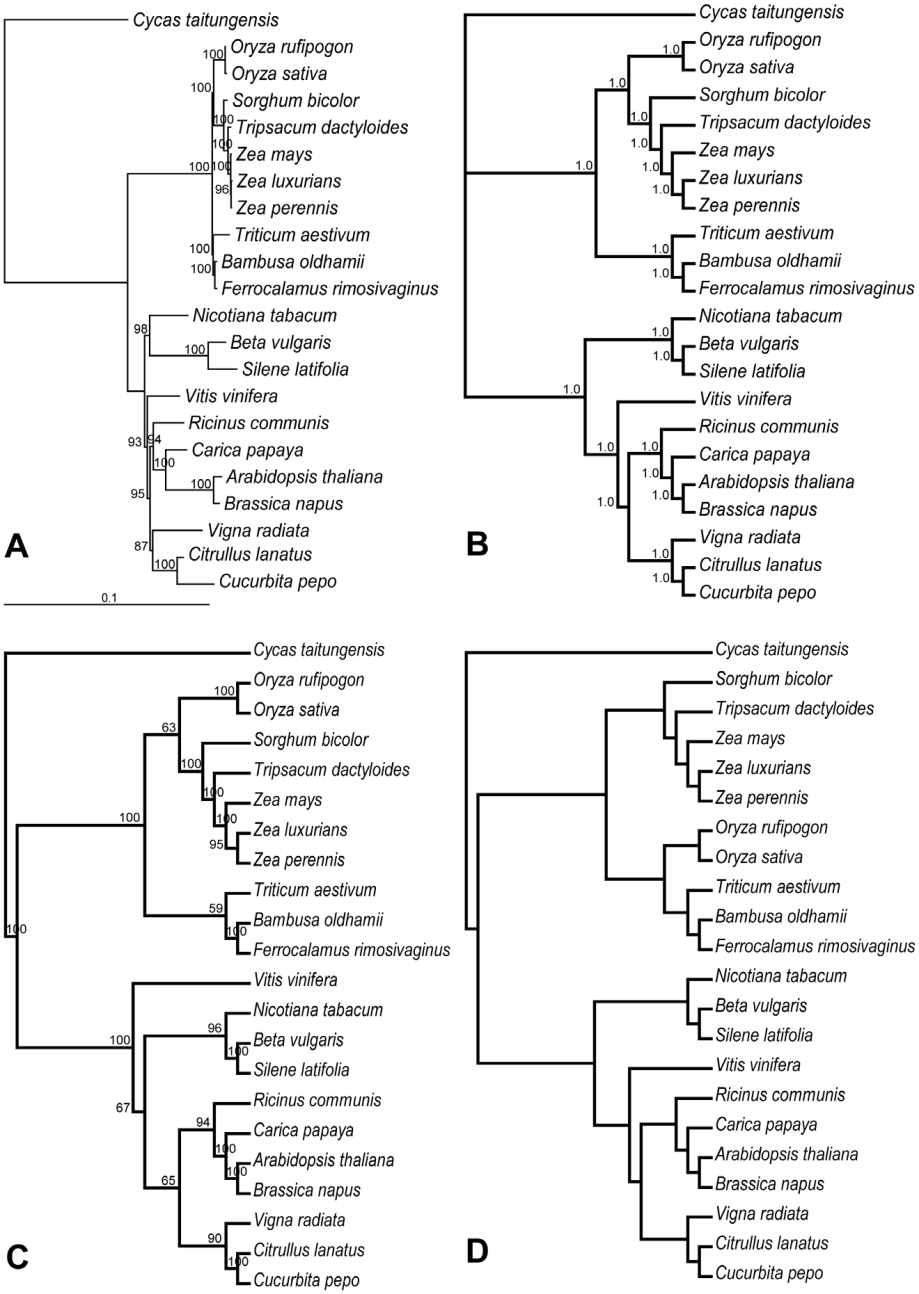
Phylogenetic trees of 22 seed plants as determined from mitochondrial and chloroplast genomes. The ML tree (A), the BI tree (B) and the MP tree (C) based on 31 mitochondrial (mt) genes. The topology of the chloroplast tree (D) is constrained by previous studies [9], [25], [42]. Numbers at nodes indicate bootstrap support (BS) values ≥50% or BI posterior probabilities. Branch lengths of the ML tree calculated through RAxML analysis, and correspond to scale bar (in units of substitutions/site).

Phylogenetic analyses were performed on a 22-taxon (Table 1), 31 mt genes of 28,728 aligned nucleotide positions using maximum likelihood (ML) method with 3 different partitioning strategies: unpartition, partitioning the data set by gene or codon position. The same tree topology and similar bootstrap support (BS) values were obtained regardless of the partitioning strategies (Figure 1A and Figure S1). To evaluate the influence of different methods imposed on phylogenetic reconstruction, maximum parsimony (MP) and Bayesian inference (BI) were also used. The BI analysis generated the same phylogenetic relationships within angiosperms as those inferred from ML and all nodes of the tree received a posterior probability of 1.0 (Figure 1B). The MP analysis resulted in a single most parsimonious tree with a length of 9,977, a consistency index (CI) of 0.64 (excluding uninformative characters), and a retention index (RI) of 0.85 (Figure 1C). The MP tree differed from the ML tree only in the placement of *Vitis vinifera*, and overall BS values were slightly lower than those of the ML tree. Furthermore, there were four nodes in the MP tree only receiving weak support (BS = 59% to 67%), two of which involved in the placement of *V. vinifera*. Below, we would focus on phylogenetic relationships with an emphasis on the ML topology.

The ML topology was well supported and phylogenetic resolutions were increased relative to a previous analysis based on only four mt genes with dense taxon sampling [29], although the taxon sampling was very different between these two studies and comparing them was not straightforward. Phylogenetic relationships inferred from mtDNA were congruent with those based on cpDNA (Figure 1A and 1D) [9], [24]–[27], [42] with the exception of one topological difference. Within Poaceae, the three subfamilies Bambusoideae (*B. oldhamii* and *F. rimosivaginus*), Ehrhartoideae (*O. sativa* and *O. rufipogon*), and Pooideae (*T. aestivum*) formed the BEP clade which had a sister relationship to the PACMAD clade (the other five grasses in the tree belong to Panicoideae) in all the previous phylogenetic analyses of the cpDNA sequences [9], [26]–[28]. However, ML analysis of mtDNA sequences did not support the monophyly of the BEP clade but a sister relationship of Ehrhartoideae+Panicoideae with 100% bootstrap support, although this sister relationship only received 63% BS support in the MP tree. Furthermore, two relative long branches (the Ehrhartoideae branch and the Panicoideae branch) separated by the short internode (Figure 1A) implied that this sister relationship might be an artifact of phylogeny reconstruction due to long branch attraction (LBA) [43], [44].

**Figure 2:**
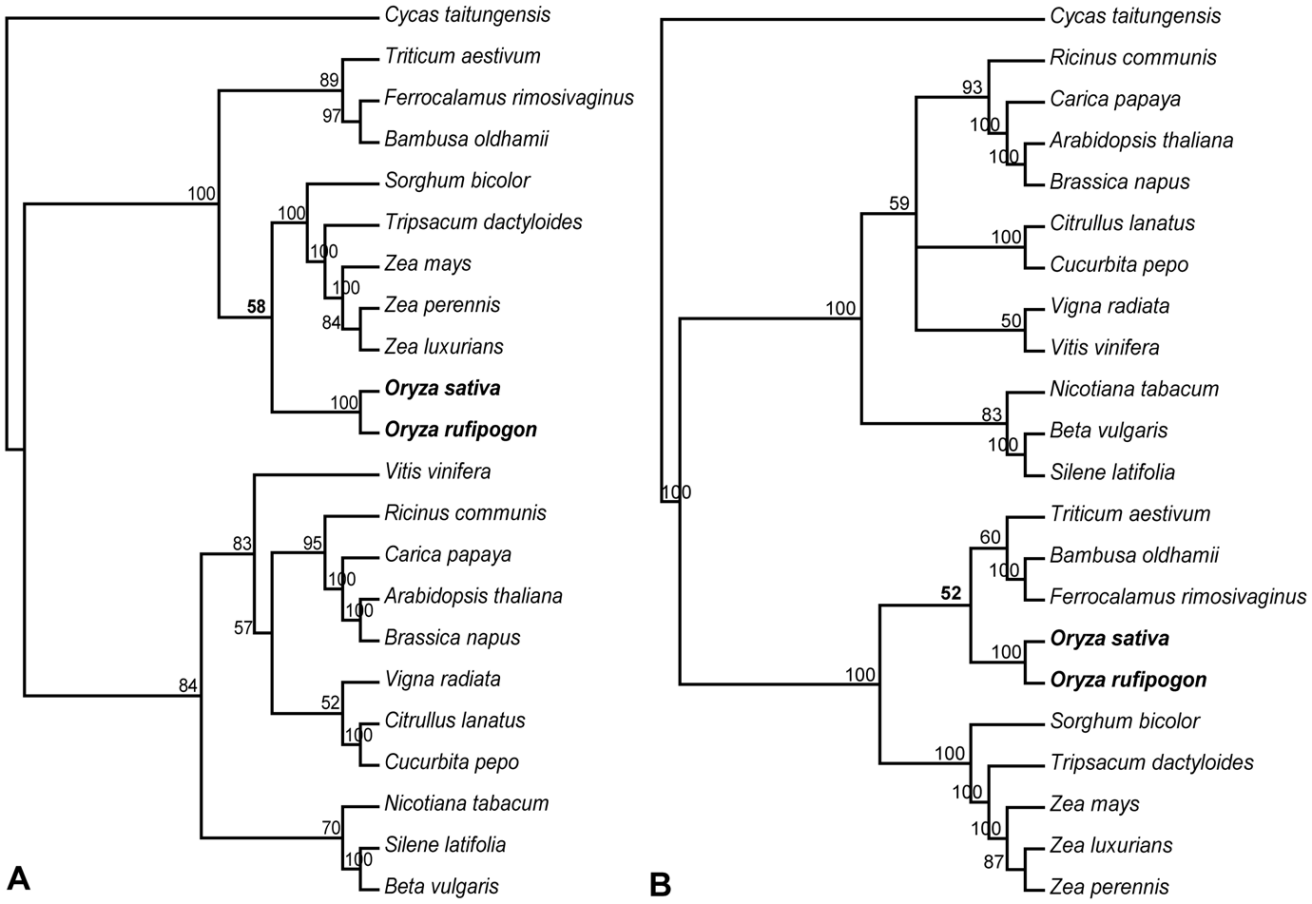
Phylogenetic trees of 22 seed plants based on amino acid sequences of 31 mitochondrial genes. The ML tree (A) and the MP tree (B). Numbers associated with branches are bootstrap support (BS) values. *Oryza* and related BS values indicated in bold.

**Figure 3:**
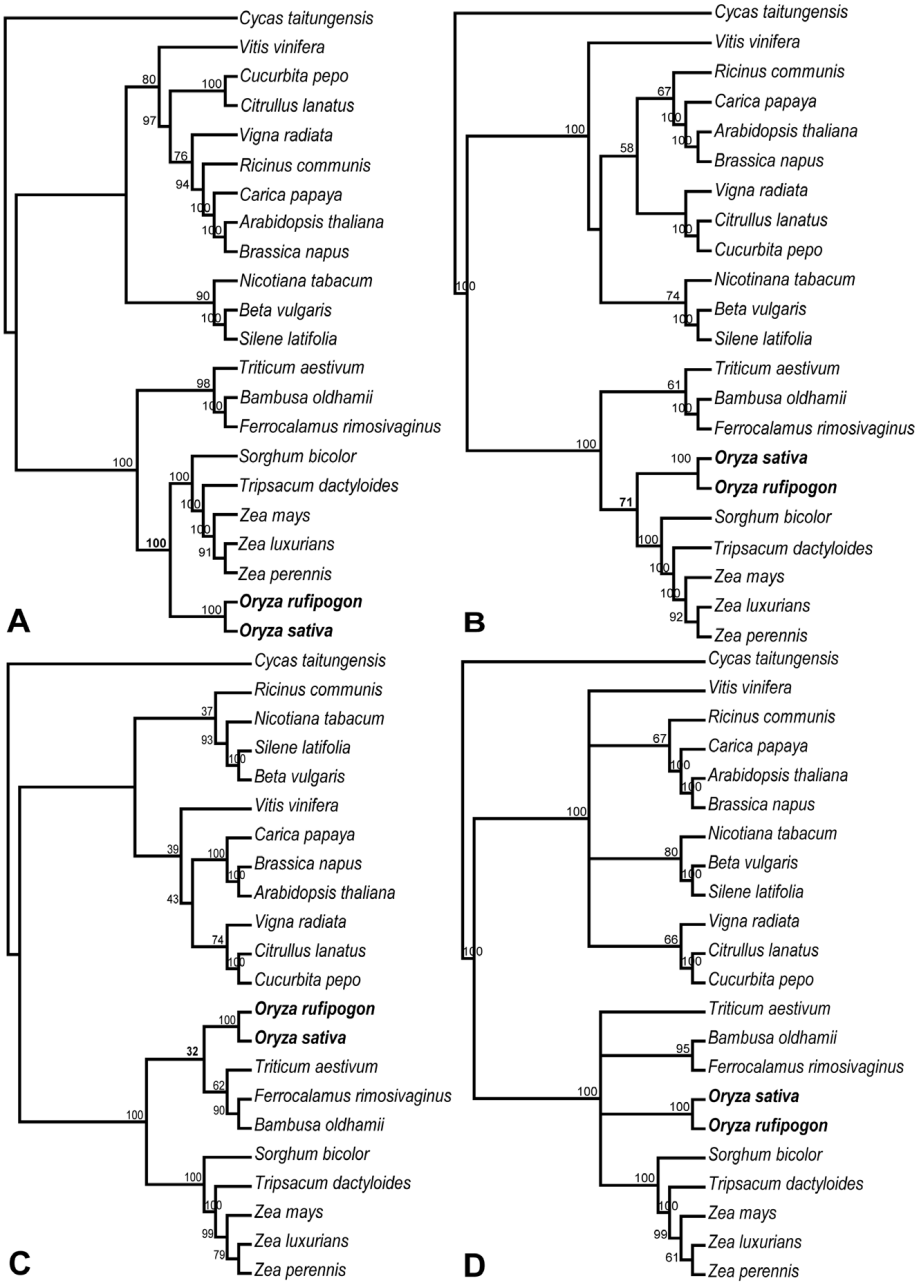
Phylogenetic trees of 22 seed plants inferred from different datasets. The ML tree (A) and the MP tree (B) inferred from the concatenated 12 fast-evolving mitochondrial (mt) genes. The ML tree (C) and the MP tree (D) inferred from the concatenated 19 slow-evolving mt genes. Numbers associated with branches are bootstrap support (BS) values. *Oryza* and related BS values indicated in bold.

To detect the LBA artifact, we first performed phylogenetic analyses at the amino acid level of the total 31 genes. The BS value for Ehrhartoideae+Panicoideae decreased dramatically from 100% to 58% in the ML tree (Figure 2A), while BS values for other nodes within Poaceae did not decrease proportionally but instead remained high (BS = 84% to 100%). On the other hand, the BEP clade was weakly supported (BS = 52%) as a monophyletic group in the MP tree (Figure 2B). We further partitioned the 31 genes into fast- and slow-evolving ones according to their substitution rates. The genes with lower substitution rate than the average of all the 31 genes (0.060 substitutions/site) were considered as slow-evolving genes and the rest as fast-evolving ones (see Materials and Methods for detail). If Ehrhartoideae+Panicoideae were an LBA artifact, then support for this group would be favored by the partitions of fast-evolving genes, whereas it would be minimized in partitions of slow-evolving genes. Consistent with our hypothesis, analyses of the concatenated 12 fast-evolving genes supported Ehrhartoideae+Panicoideae with 100% BS value in the ML tree (Figure 3A), and BS value for this group in the MP tree increased from 63% based on all the 31 genes to 71% (Figure 3B and Figure 1C). Furthermore, the monophyly of the BEP clade (BS = 32%) was recovered in ML analysis of the concatenated 19 slow-evolving genes (Figure 3C), and the relationships of the BEP clade within Poaceae were not resolved in MP analysis of this data (Figure 3D).

Rare genomic changes such as gene losses, arrangements of genes, insertions and deletions of introns are less prone to homoplasy than nucleotide sequences and have become an alternative approach for phylogenetic studies [45], [46]. Two gene losses, *rpl5* and *rps19*, were restricted to the mt genomes of Panicoideae and pseudogene of *rps14* was only retained in the mt genomes of the BEP clade (Table 4). These rare genomic changes supported the monophyly of the BEP clade as well.

### Pattern of Rate Change in Grass Mitochondrial Genomes

The long branch leading to Poaceae implied that the mt genomes of this family may undergo an elevated evolutionary rate (Figure 1A), just like the cp genomes of this family [22], [31], [47]. To quantify the evolutionary rate in grass mt genomes, we calculated absolute substitution rates (R) in substitutions per site per billion years (SSB) as described by Parkinson et al. [18] in mtDNA tree with the placement of *Oryza* constrained by Figure 1D. The values of R for branches involving Poaceae were calculated by dividing the branch length by the length of time for that branch. Divergence times were based on estimates in previous studies [25], [32]–[34], with separation of monocots/eudicots and origin of core Poaceae setting at 135 Myr and 65 Myr, respectively (other divergence times in Table S3). Because there was no reliable estimate of divergence times within genera *Oryza* and *Zea*, R was averaged among them from the terminal species to the common ancestor of *Oryza*/*Triticum* or *Zea*/*Tripsacum*.

**Figure 4:**
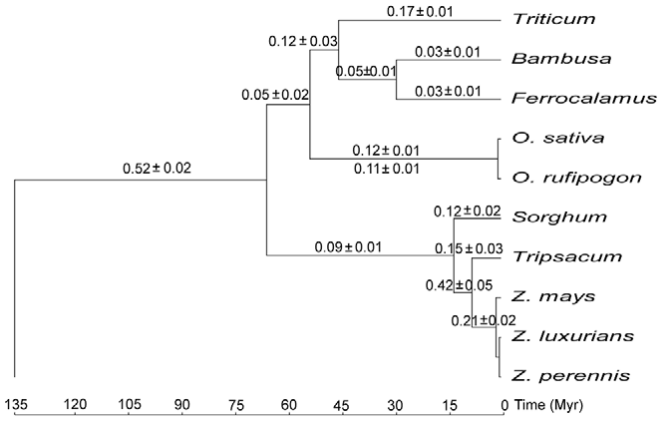
Absolute substitution rates during the evolutionary history of grasses. Values above each branch indicate absolute substitution rate (R) in substitutions per site per billion years (SSB) for that branch. Among them, 0.12±0.01 and 0.11±0.01 above and below the branch to *Oryza* are the mean values along the lineage from the common ancestor of *Oryza*/*Triticum* to *O. sativa* and *O. rufipogon*, respectively, while 0.21±0.02 above the branch to *Zea* is the average value of R along the lineage from the common ancestor of *Zea*/*Tripsacum* to three *Zea* species.

Figure 4 illustrated that the evolutionary rate of mt genome changed many times during grass evolution. Comparison of the fastest and slowest lineages showed that the rate varied by a factor of 17. The elevation of rate occurred on the common branch of Poaceae after they diverged from eudicots, and the majority of rates after diversification of Poaceae were ∼4-fold slower than that on the branch leading to Poaceae. For example, the rate along the lineages from monocots/eudicots separation to *O. sativa* changed from 0.52 to 0.05 to 0.12 SSB. This pattern of rate change resembled that observed in grass cp genomes [22]. Another notable feature of Figure 4 was the lower evolutionary rate in the lineages of Bambusoideae compared to the other three subfamilies in Poaceae. To examine the impact of divergence times on demonstrating rate change, we also applied much older divergence times 212 Myr and 72 Myr used in estimating evolutionary rate in grass cp genomes [22] for monocots/eudicots separation and origin of core Poaceae, respectively. Nevertheless, the same tendency for rate change in mt genome was obtained with these calibration points (Figure S2).

To check the rate decrease of mt genome after diversification of Poaceae in more detail, we partitioned the substitutions into synonymous and nonsynonymous ones. The same pattern of rate change as that of total substitutions was observed in both types of substitutions (Figure S3). And nonsynonymous/synonymous rate ratio (ω) on the branch leading to Poaceae was 0.42, indicating overall purifying selection operating on these genes during grass evolution. However, we did not exclude the RNA editing sites of mt genes in calculating ω and the existence of RNA editing sites can bias the estimate of it [48].

**Figure 5:**
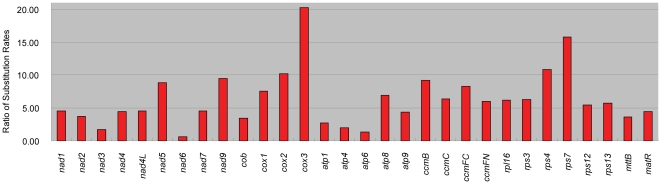
Rate change variation among genes in Poaceae. Each bar represents the ratio of absolute substitution rate (R) along the line leading to Poaceae to that on the line from the common ancestor of grasses to *O. sativa*.

The evolutionary rates above were estimated on the concatenated 31-gene data set. However, different genes of mt genome in the same plant lineage could have various evolutionary rates [16], [19]. To explore the rate change in individual genes, the ratio of R before diversification of Poaceae to that on the line from the common ancestor of Poaceae to *O. sativa* for each gene was calculated (Figure 5). Among them two genes, *nad4L* and *nad7*, had no nucleotide substitutions during the period from Poaceae origin to *O. sativa* and thus the ratios for them were artificially given the average value 6.46 for all the other genes. All the genes except for *nad6* showed the same pattern of rate change of elevated rate before diversification of Poaceae with subsequent slow-down after diversification (Figure 5) and this pattern was consistent with that based on analysis of combined genes. Furthermore, the rate change exhibited limit of variation between genes and the majority of ratios had a value around 5.00.

## Discussion

Due to their high-throughput and low-cost, next-generation sequencing technologies have greatly improved the approaches for genome sequencing. For angiosperm organelle genomes, however, they have been largely restricted to sequencing of cp genomes until now. Using next-generation sequencing platforms for mt genome sequencing has only recently been explored [6]–[8]. Furthermore, only a few angiosperms have had their mt genomes sequenced, and more completed genomes are necessary to study the evolution of angiosperm mt genomes. Here we present a largely completed mt genome from the bamboo *F. rimosivaginus* mainly based on Illumina sequencing, providing the demonstration of the feasibility for sequencing angiosperm mt genomes with Illumina sequencing technique. With successful sequencing of the mt genomes using Illumina and 454 sequencing technologies [6]–[8] it has become evident that high-throughput next-generation sequencers could hold promise for the angiosperm mt genomes sequencing in the near future. The Illumina sequence reads for the other five bamboos in [9] are under analysis and similar results are obtained. This rapid and effective approach for the bamboo *F. rimosivaginus* mt genome sequencing could be an alternative to the established methods for angiosperm mt genomes sequencing.

### Feasibility of Illumina Sequencing of Angiosperm Mitochondrial Genomes

Given the highly variable sizes of angiosperm mt genomes [12], [49], [50], we could only deduce that the *F. rimosivaginus* mt genome were largely completed based on mean size of mt genomes from three closely related grasses. Nevertheless, our assembly appears to be 100% complete with regard to gene content, as the housekeeping genes shared by the reference mt genomes are all identified from our assembly. Additionally, the draft genome is of relatively high quality with a N50 size of 53.1 kb and only 14 gaps remained to be finished to complete the genome. However, it should be noted that the availability of closely related reference genomes in the grass family and presumed less large repeats in bamboo mt genomes [51] may have made it relatively easier to assemble than some other angiosperm mt genomes.

In compared to angiosperm cp genomes, it would be much difficult to assemble mt genome from Illumina short reads because of larger genome size, more repetitive sequences and frequent genome rearrangements. For assembly of the *F. rimosivaginus* mt genome, there are three probable reasons that could explain why we have not obtained a single, full-length genome sequence. First, the raw reads just do not represent complete coverage of the genome. The sequencing depth could vary throughout the genome due to GC content or other factors [52]. However, in light of the relatively high 39.5× average sequencing depth, we argue that the raw reads may have covered the whole genome. Second, it is failure to retrieve certain assembled contigs and/or scaffolds whose sequences are actually derived from the *F. rimosivaginus* mt genome during assembly. However, the availability of several closely related reference genomes and the low threshold used in mapping contigs and scaffolds (see details in Materials and Methods) would dramatically reduce this possibility. In fact, about 17.9% of the assembled sequences have no similarity to the sequences of any other sequenced mt genomes of plants as well as the sequences in the NCBI non-redundant nucleotide and protein databases (data not shown). Third, the incorporated sequences from cpDNA of *F. rimosivaginus* in the mt genome were excluded from the assembly. The reported uptakes of cpDNA sequences by angiosperm mt genomes constitute 1.1–11.5% of the genome size [49], [53]. These cp-derived sequences are very likely to affect our assembly and thus we removed assembled sequences with significant sequence identity to the cp genome of *F. rimosivaginus*. This is a potential disadvantage of our approach for mt genome sequencing. Among three explanations above, the last one may contribute mostly to the incompleteness of our assembled genome. In the future, modifying the method used in DNA extraction to reduce the proportion of cpDNA in the total DNA could solve this problem. Advances in next-generation sequencing technologies, such as increase in length and number of sequence reads (more coverage) and paired-end sequencing with larger insert size, will also improve the assembly.

### Gene Content of the *F. rimosivaginus* Mitochondrial Genome

The mt genome of *F. rimosivaginus* is the second sequenced mt genome from a bamboo species in Poaceae. It encodes roughly the same protein genes as mt genomes of other grasses, with the loss of *sdh3*, *sdh4* and several ribosomal protein genes which are all prone to loss during angiosperm evolution [11]. An *rpl14* pseudogene is retained in the genome in good agreement with survival of the pseudogene in grasses reported before [41]. Like other grass mt genomes, the *F. rimosivaginus* mt genome does not encode the full set of tRNAs necessary to recognize all codons, and nearly half of them are cp-derived tRNAs. The mt genome contains the identical gene set for tRNAs of mt origin among the ten sequenced grasses, while their genomes do not share the same tRNAs of cp origin, indicating an ongoing process of acquisition and loss of cp-derived tRNAs.

### Parallel Episodic Evolution of Organelle Genomes

At present, phylogenies of angiosperms are essentially reconstructed from the cpDNA sequences. If the cp and mt genomes are both strictly maternally inherited in angiosperms, we would expect them to contain identical phylogenetic signals. As expected, the relationships within our reconstructed mtDNA tree were largely congruent with phylogenetic analyses of the cpDNA sequences [9], [24]–[27], [42]. The well-supported topology confirms the utility of mtDNA for phylogenetic reconstruction [29], [30] and mt phylogenomics may be useful for resolving some difficult angiosperm phylogenies. In spite of overall congruence, the angiosperm mtDNA tree is in obvious contrast to cpDNA tree in the monophyly of the BEP clade. Using more conserved protein sequences of mt genes, the monophyly or significant decrease in support for non-monophyly of the BEP clade was obtained in mtDNA tree. Furthermore, the fast-evolving genes were more favored than the slow-evolving genes in non-monophyly of the BEP clade. Genome-level features of mt genome that are less subjective to homoplasy also support the monophyly of the BEP clade. In summary, these results suggest that the sister relationship of Ehrhartoideae+Panicoideae inferred from mtDNA is most likely an LBA artifact. The fast-evolving genes which have high different evolutionary rates among different lineages within Poaceae may largely contribute to the LBA artifact [54], [55]. In addition, the taxon sampling in our phylogenetic analyses was very sparse. Thus, LBA due to poor taxon sampling [43], [44] may also result in this phylogenetic inconsistency.

Unlike prior studies based on a few mt genes [15]–[19], we used nearly all the protein genes of mt genome to demonstrate episodic evolution of mt genome in grasses. Moreover, all the examined genes except for *nad6* had the same pattern of rate change. Although completed mt genomes of other monocots are not available to break the long, poorly sampled branch leading to Poaceae, evolutionary rate averaging along the branch is still faster than those after diversification of Poaceae. However, the range of rate variation is much smaller than those recently documented [16]–[19]. And in contrast to rate variations being restricted to synonymous substitutions in these studies, nonsynonymous substitutions in grass mt genomes also show the corresponding rate change. The factors that are responsible for this rate change are very likely to be different from those with locus effect proposed before, such as the efficiency of mtDNA repair. Furthermore, that the episodic evolution is correlated between the mt genome and the cp genome in grasses is consistent with lineage effects. The same underlying factors for the rate change may simultaneously operate on the mt and cp genomes of grasses. However, we could not confirm the rate acceleration of mt genome only occurring in the ancestral grasses like that of cp genome with the present data. Completed mt genomes from taxa closely related to Poaceae are required to resolve this question in further studies.

## Materials and Methods

### Mitochondrial DNA Isolation, Genome Sequencing and Assembly

Total DNA enriched for cpDNA was extracted and sequenced using the protocols described in [9]. The raw short reads were firstly assembled using SOAPdenovo [35] with *K* = 31 bp and scaffolding contigs with a minimum size of 100 bp. All the assembled scaffolds and contigs (≥100 bp) were mapped to the sequenced mt genomes in Poaceae (Table 1) using BLASTN searches from NCBI with default parameters. Those contigs and scaffolds whose query coverage was greater than 40% were retrieved and then they were used to search against the NCBI non-redundant nucleotide and protein databases with BLASTN (http://blast.ncbi.nlm.nih.gov/). These without significant identity to sequences from the cp and/or nuclear genomes were deemed as sequences derived from mtDNA. In this procedure, we removed 3 scaffolds and 35 contigs which could be aligned to reference mt genomes but supposed to be of cpDNA origin as they had significant sequences identity (≥90%) to the mt genomes as well as the *F. rimosivaginus* cp genome. We realigned all the raw reads onto the assembled sequences using software Bowtie [56] with −v = 3. The aligned paired-end reads were used to determine the sequencing depth. A second round of assembly was carried out on the initially assembled contigs and scaffolds. By using the information of overlap of ≥12 bp between contigs and scaffolds and synteny between assembled and reference genomes, we further joined them into larger scaffolds.

### PCR-Based Genome Finishing and Validation

Following the steps above, the candidate linkages of the contigs and/or scaffolds were validated by PCR analysis. We designed primers according to the nucleotide sequences surrounding the linkage and in all 12 primer pairs were used. To close the gaps inside the scaffolds, 20 primer pairs were also designed. Furthermore, we randomly chose 16 regions in the assembly for resequencing using PCR. All the primer sequences were in Table S4. PCR products were sequenced by ABI 3730xl genetic analyzer using standard protocols. Sanger sequences and the assembly were aligned using MEGA 4.0 [57] to determine if there were any differences.

### Genome Annotation

A preliminary annotation was carried out by mapping BLASTN hits employing known mt genes of grasses as queries and subsequently, by testing for consistency of the open reading frame. The exact gene and exon boundaries were determined by alignment of homologous genes from *B. oldhamii*. We also used tRNAscan-SE 1.21 [58] to corroborate tRNA boundaries identified by BLASTN. The sequences of identified tRNA genes were BLAST searched against the cp genome of *F. rimosivaginus* to detect cp-derived tRNA genes.

### Phylogenetic Analyses

We extracted nucleotide sequences for all protein genes from 22 seed plant mt genomes (Table 1). After excluding several genes (*sdh3*, *sdh4*, *rpl2*, *rpl6*, *rps1*, *rps2*, *rps8*, *rps10*, *rps11*, *rps14*, and *rps19*) that are missing from many of the sequenced genomes, 31 genes were retained. Each gene was aligned separately with MEGA 4.0 constrained by its amino acid sequence. Alignments of nucleotide sequences were then manually adjusted, and ambiguously aligned regions were excluded from the analysis. We did not exclude RNA editing sites as several studies suggested that they were not a problem in phylogenetic reconstruction, especially with large sequence data sets [29], [30], [59], [60]. Finally, individual genes were concatenated, and the resulted alignment consisted of 28,728 nucleotides.

ML analyses of the 31-gene data set were performed with RAxML 7.0.4 [61] using three partitioning strategies: unpartitioned, partitioned the data based on gene region, and partitioned the protein genes by each codon position. All ML analyses used the rapid BS algorithm implemented in RAxML 7.0.4 with 1,000 replicates and the general time-reversible (GTR) model of evolution with among-site rate variation. For BI, we used software MrBayes 3.1.2 [62] with GTR+G+I model. The run started with a random tree, default priors, and four Markov chains, totalling 1 million generations with sampling trees every 100th generation. When convergence was obtained, a consensus tree was calculated after omitting the first 25% of trees as burn-in. For MP, we used PAUP*4.0b10 [63] to implement heuristic searches that consisted of TBR branch swapping, starting from 1,000 trees built by random taxon stepwise addition, and multrees option in effect. Non-parametric bootstrap analysis was conducted under 200 replicates with TBR branch swapping from 100 random taxon addition staring trees. To detect long branch attraction, phylogenetic trees were constructed from the amino acid sequences of 31 genes with both ML and MP analysis conducted like before. ML analysis of protein sequences was performed with the Dayhoff matrix of amino acid substitutions and a discrete gamma distribution with four rate categories. The substitution rates (in number of substitutions per site) of the 31 genes in the 22 sampled species were calculated by MEGA 4.0 under the model of Kimura 2-Parameter, ranging from 0.027 to 0.125 with a mean of 0.060. Nineteen of the 31 genes (*nad1*, *nad2*, *nad3*, *nad4*, *nad4L*, *nad5*, *nad6*, *nad7*, *nad9*, *cob*, *cox1*, *cox3*, *ccmB*, *ccmC*, *rpl16*, *rps7*, *rps12*, *rps13*, and *mttB*) with substitution rates lower than the average were concatenated to form the 19 slow-evolving genes data and the rest (*cox2*, *atp1*, *atp4*, *atp6*, *atp8*, *atp9*, *ccmFC*, *ccmFN*, *rpl5*, *rps3*, *rps4*, and *matR*) formed the 12 fast-evolving genes data. ML and MP analyses were also performed on the concatenated 12 fast-evolving genes and 19 slow-evolving genes in the same way.

### Estimation of Absolute Substitution Rates

Absolute substitution rates were calculated for the Poaceae lineages using method that has been described before [18]. Briefly, branch lengths that represent the number of substitutions per site were determined for concatenation of 31 genes using codeml in PAML v4.4 [64] with topologically constrained tree. Codon frequencies were computed by using F3×4 method and separate ω ratios were estimated for each branch. Relationships within the tree were based on Figure 1D. Divergence times used (Table S3) were taken from previous studies [25], [32]–[34]. Then absolute substitution rate per branch was calculated by dividing the branch length by the length of time for that branch. Standard errors were determined as in [18]. Absolute substitution rates in terms of nonsynonymous and synonymous substitutions were calculated in the same way. For 30 individual genes (*rpl5* was excluded for loss in Panicoideae), the absolute substitution rate was analyzed in the same manner as that of concatenated genes and then the ratio of absolute substitution rate before diversification of Poaceae to from origin of the common ancestor of Poaceae to *O. sativa* was calculated.

## Supporting Information

Figure S1 Phylogenetic trees as determined by RAxML based on the 31 mitochondrial genes, under the following partitioning schemes: A) partitioned by gene; B) partitioned by codon position. Numbers at nodes indicate bootstrap support (BS) values.
(TIF)

Figure S2 Rate changes in grass mitochondrial genes during evolution with divergence times 212 Myr and 72 Myr for monocots/eudicots separation and origin of core Poaceae, respectively.
(TIF)

Figure S3 Rates of nonsynonymous (A) and synonymous (B) substitutions changes in grass mitochondrial genes during evolution.
(TIF)

Table S1 Lists of assembled initial and final contigs and scaffolds.
(XLS)

Table S2 Comparison of the assembly and Sanger sequences.
(XLS)

Table S3 Divergence times used in calculating absolute substitution rates of grass mitochondrial genes.
(DOC)

Table S4 Information on the primers used for the PCR analyses to validate the linkage between the contigs and/or scaffolds and close intra-scaffold gaps.
(XLS)
