# Supplementary figures and images for: Rapid Sequencing of the Bamboo Mitochondrial Genome Using Illumina Technology and Parallel Episodic Evolution of Organelle Genomes in Grasses
Source: PLoS One. 2012 Jan 17;7(1):e30297. doi: 10.1371/journal.pone.0030297 (PMC3260276; doi:10.1371/journal.pone.0030297)

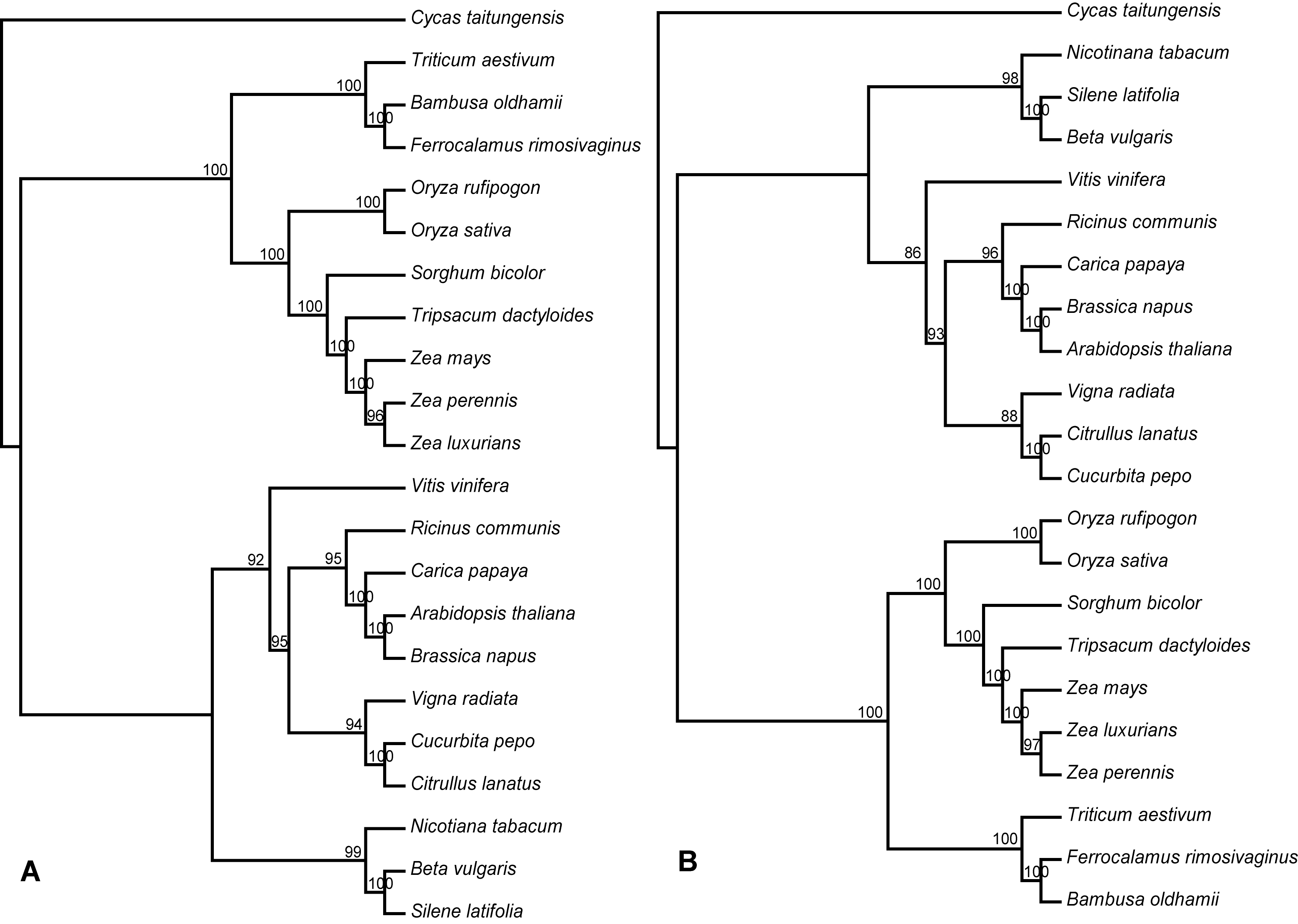

Supplement: Figure S1 — Phylogenetic trees as determined by RAxML based on the 31 mitochondrial genes, under the following partitioning schemes: A) partitioned by gene; B) partitioned by codon position. Numbers at nodes indicate bootstrap support (BS) values. (TIF) [file pone.0030297.s001.tif]

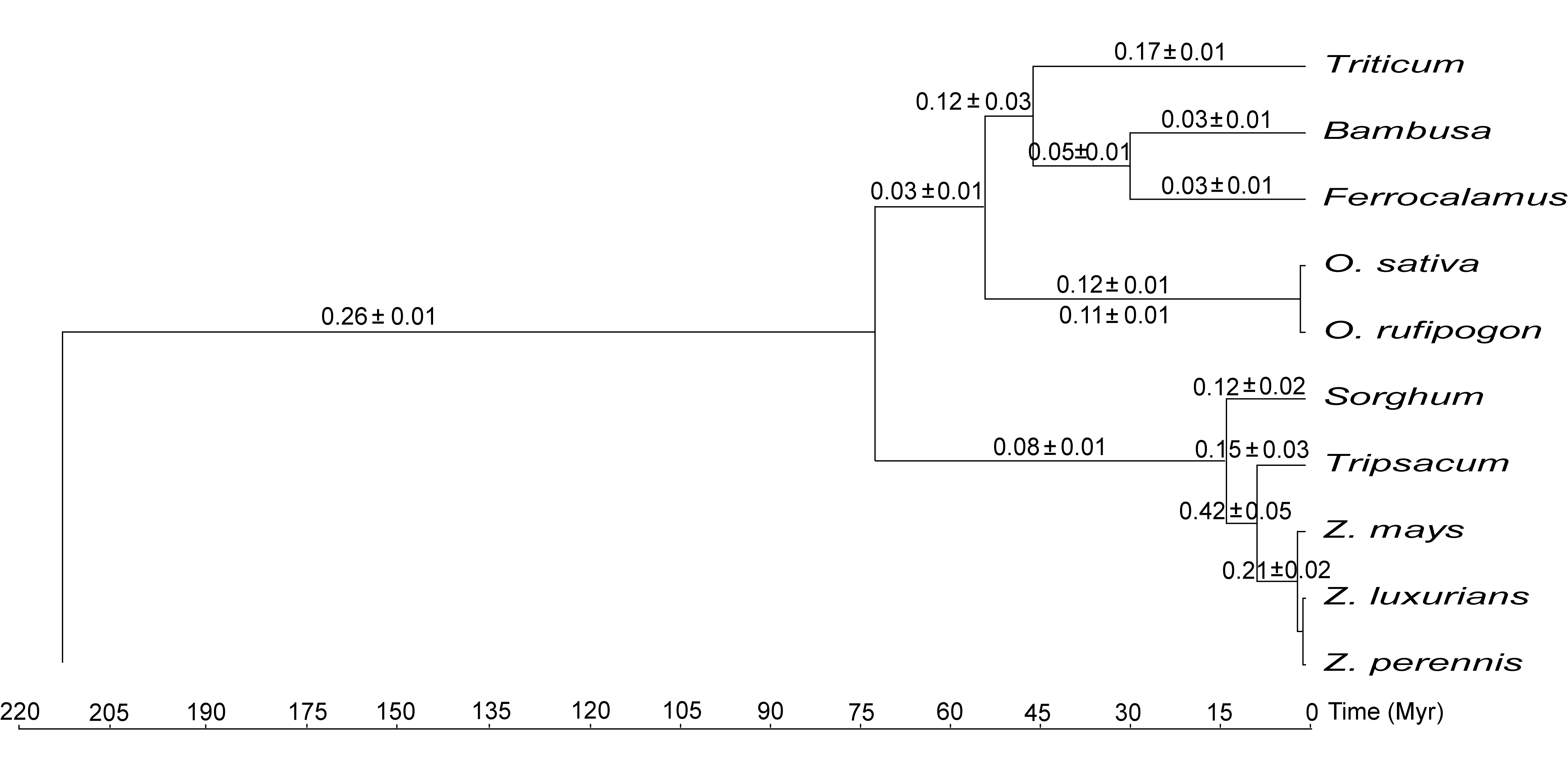

Supplement: Figure S2 — Rate changes in grass mitochondrial genes during evolution with divergence times 212 Myr and 72 Myr for monocots/eudicots separation and origin of core Poaceae, respectively. (TIF) [file pone.0030297.s002.tif]

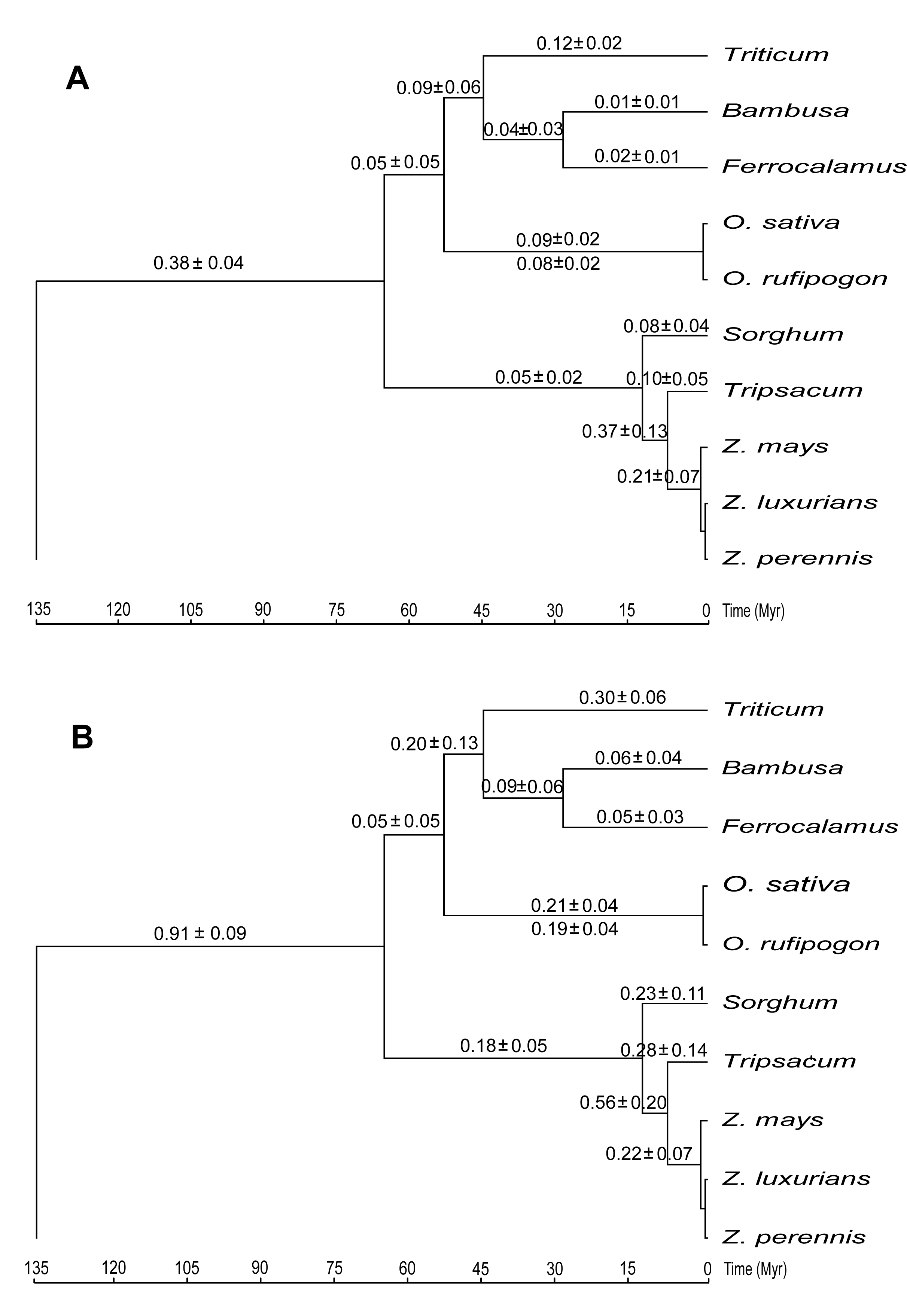

Supplement: Figure S3 — Rates of nonsynonymous (A) and synonymous (B) substitutions changes in grass mitochondrial genes during evolution. (TIF) [file pone.0030297.s003.tif]
