# Supplementary material for: Rapid Sequencing of the Bamboo Mitochondrial Genome Using Illumina Technology and Parallel Episodic Evolution of Organelle Genomes in Grasses
Source: PLoS One. 2012 Jan 17;7(1):e30297. doi: 10.1371/journal.pone.0030297 (PMC3260276; doi:10.1371/journal.pone.0030297)
Supplement: Table S3 — Divergence times used in calculating absolute substitution rates of grass mitochondrial genes. (DOC) [file pone.0030297.s006.doc]

**Table S3 Divergence times used in calculating absolute substitution rates of grass mitochondrial genes.**

| Separation | Time (Myr) | Reference |
| --- | --- | --- |
| monocots/eudicots | 135 | Moore et al. [25] |
| Origin of core Poaceae | 65 | Piperno and Sues [32] |
| *Triticum*/*Oryza* | 53 | Bouchenak-Khelladi et al [34] |
| *Triticum*/*Ferrocalamus* | 45 | Bouchenak-Khelladi et al [34] |
| *Bambusa*/*Ferrocalamus* | 29 | Bouchenak-Khelladi et al [34] |
| *Sorghum*/*Zea* | 13 | Vicentini et al. [33] |
| *Tripsacum*/*Zea* | 8 | Vicentini et al. [33] |

Note: References are cited in the main manuscript document.
